# Differences in SARS-CoV-2 Clinical Manifestations and Disease Severity in Children and Adolescents by Infecting Variant

**DOI:** 10.3201/eid2811.220577

**Published:** 2022-11

**Authors:** Ana Maria Quintero, Mariah Eisner, Rouba Sayegh, Tori Wright, Octavio Ramilo, Amy L. Leber, Huanyu Wang, Asuncion Mejias

**Affiliations:** Nationwide Children’s Hospital, Columbus, Ohio, USA (A.M. Quintero, R. Sayegh, T. Wright, O. Ramilo, A.L. Leber, H. Wang, A. Mejias);; Biostatistics Resource at Nationwide Children’s Hospital, Columbus (M. Eisner);; The Ohio State University, Columbus (M. Eisner, O. Ramilo, H. Wang, A. Mejias)

**Keywords:** COVID-19, SARS-CoV-2, coronavirus disease, severe acute respiratory syndrome coronavirus 2, respiratory infections, zoonoses, viruses, variants, children, disease severity, United States

## Abstract

Since the COVID-19 pandemic began, different SARS-CoV-2 variants have been identified and associated with higher transmissibility than the ancestral nonvariant strain. During January 1, 2021–January 15, 2022, we assessed differences in clinical and viral parameters in a convenience sample of COVID-19 outpatients and inpatients 0–21 years of age in Columbus, Ohio, USA, according to the infecting variant, identified using a mutation-specific reverse transcription PCR assay. Of the 676 patients in the study, 17.75% were infected with nonvariant strains, 18.49% with the Alpha variant, 41.72% with Delta, and 16.42% with Omicron. Rates of SARS-COV-2/viral co-infections were 15.66%–29.41% and were comparable across infecting variants. Inpatients with acute Delta and Omicron infections had lower SARS-CoV-2 cycle threshold values and more frequent fever and respiratory symptoms than those with nonvariant strain infections. In addition, SARS-COV-2/viral co-infections and the presence of underlying conditions were independently associated with worse clinical outcomes, irrespective of the infecting variant.

SARS-CoV-2, the etiologic agent of COVID-19, rapidly spread worldwide, causing a global pandemic with major social and economic disruption. Although the effects of COVID-19 have been greater in adults, children also are infected with SARS-CoV-2, and COVID-19 can lead to severe outcomes in pediatric patients (*1*–*3*). Nevertheless, the spectrum of clinical manifestations in children is broad and ranges from asymptomatic to mild upper respiratory infection to pneumonia or the more severe multisystem inflammatory syndrome in children (MIS-C), which typically occurs 2 to 6 weeks after acute SARS-CoV-2 infection (*4*–*7*).

Since the COVID-19 pandemic began, different SARS-CoV-2 variants have circulated worldwide. In the United States, the first variant that replaced the original strain was the Alpha variant (B 1.1.7) that circulated during April–June 2021. The Delta variant (B1.617.2) followed soon after and became predominant during July–mid-December 2021. Since then, different sublineages of Omicron quickly replaced other variants as the predominant variant as of September 2022. These newer variants have demonstrated higher transmissibility and have disproportionally affected unvaccinated persons and other vulnerable populations including children; rates of hospitalization have increased 5-fold to 10-fold in children, depending on the variant and age group (*8*–*13*). Epidemiologic studies that rely on SARS-CoV-2 circulation patterns have provided robust information; however, the role of specific SARS-CoV-2 variants on clinical disease severity in children and adolescents with COVID-19 is not fully known.

The objective of this study was to assess whether distinct SARS-CoV-2 variants were associated with differences in clinical and laboratory data and cycle threshold (Ct) values (as a surrogate of viral load) in children and adolescents with COVID-19. The Nationwide Children’s Hospital (NCH; Columbus, OH, USA) Institutional Review Board approved the study (#STUDY00002002).

## Methods

### Sample Collection and Testing Algorithm

During January 1, 2021–January 15, 2022, we identified nasopharyngeal (NP) samples from children and adolescents <21 years of age that tested positive by various nucleic acid amplification tests (NAATs) for SARS-CoV-2 at the Clinical Microbiology Laboratory at NCH, per standard of care (Appendix). Samples positive for SARS-CoV-2 by any of the NAATs assays were stored at −20°C.

From all available specimens, we selected a convenience sample for variant screening within 1 week of storage based on the clinical laboratory testing capability, sample volumes, and Ct values, considering a sample adequate when Ct values were <35. We used Ct values as a proxy for viral load quantification because they have an inverse relationship with quantitative viral loads (*14*).

### SARS-CoV-2 Variant Testing

We screened SARS-CoV-2–positive samples by mutation-specific reverse transcription PCR assays for Alpha, Beta, Gamma, Omicron, and other variants of interest as described (*15*). We developed a T487K assay for screening of the Delta variant (Appendix).

We considered samples positive for P1 but not P2 to be negative for the T478K mutation, and samples positive for both P1 and P2 to be positive for the T478K mutation. We designated samples that carried both L452R and T478K mutations as the Delta variant. Because the Omicron variant appeared in the United States in December 2021 when the Alpha variant had effectively disappeared (*16*), we designated samples collected during December 1, 2021–January 15, 2022, that were positive for Δ69/70 and negative for the L452R mutation as Omicron (B.1.1.529).

### Patient Selection and Data Collection

We linked identifiers from outpatients and inpatients whose samples underwent SARS-CoV-2 variant screening with their electronic healthcare records (EHRs), extracted pertinent data, and manually reviewed clinical data. We included in the inpatient cohort 1 patient who tested positive as outpatient but eventually required hospitalization within 4 weeks of diagnosis; for this patient, we considered for analyses the first sample obtained. For patients with multiple positive SARS-CoV-2 tests during the study, we included in the analyses the first sample collected and the data related to the first encounter. We considered subsequent samples collected for an individual patient or subsequent admissions to be duplicates and excluded them from analysis.

We described demographic characteristics including underlying conditions, the infecting variant type, and SARS-CoV-2 Ct values for the COVID-19 clinical cohort comprised of outpatients and inpatients; we analyzed clinical manifestations, laboratory parameters, and clinical outcomes exclusively in inpatients with acute COVID-19. We grouped underlying conditions into categories including respiratory, neurologic, genetic, immunocompromised conditions, renal/gastrointestinal, endocrine, and hematologic diseases. We also included obesity, defined as presence of age-sex-standardized body mass index z-scores >95th percentile, and overweight, defined as presence of age-sex-standardized body mass index z-scores >85th percentile; these values were based on weight (measured at the time of SARS-CoV-2 testing) and height registered in the EHR within 60 days of cohort entrance. For children <2 years of age, we determined the nutritional status by z-scores according to weight-for-age and weight-for-height, considering overweight as 1.0 to <2.0 SD and obesity as >2 SD. We grouped obesity and overweight as a single variable during data collection. To contrast the prevalence of underlying conditions between the COVID-19 clinical cohort and the patient population evaluated at NCH during the same period, we used the Pediatric Medical Complexity Algorithm (PMCA) version 2.0, which categorized patients as having no chronic conditions, noncomplex chronic conditions, or complex chronic condition comorbidities (*17*).

### Statistical Analysis

We used descriptive analysis to summarize patients’ characteristics. We analyzed categorical variables by χ^2^ or Fisher exact tests and expressed them in frequencies and percentages. We analyzed continuous variables by Kruskal-Wallis rank-sum test and expressed them as median (interquartile range) because data were nonnormally distributed. We conducted multivariable analyses to identify risk factors associated with clinical outcomes in children and adolescents with acute COVID-19, including the need for hospitalization and, in inpatients, oxygen administration and pediatric intensive care unit (PICU) admission. We built statistical models using logistic regression; in all models, the primary exposure was the infecting variant. Other covariates included were age, underlying conditions, Ct values, and viral co-infections. We evaluated models for collinearity using the generalized variance inflation factor. We performed statistical analyses in R version 4.0 (The R Project for Statistical Computing, https://www.r-project.org) and Prism version 9.0 (GraphPad Software, https://www.graphpad.com) and considered 2-sided p<0.05 statistically significant.

## Results

### Shifts in the Circulation of SARS-CoV-2 Strains

**Figure 1:**
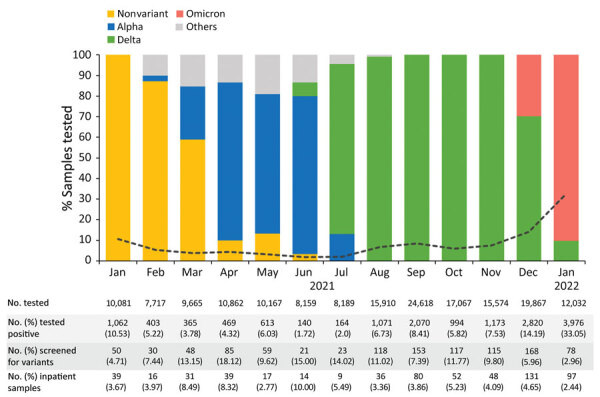
Shifts in the circulating SARS-CoV-2 variants identified at Nationwide Children’s Hospital, Columbus, Ohio, USA, by percentage of total cases irrespective of patient age, January 2021–January 2022. The others category comprises Beta (n = 12), Iota (n = 9), Zeta (n = 7), Eta (n = 2), Epsilon (n = 3), and Mu (n = 2) variants, as well as variants under investigation (n = 2). The black dotted line represents the rate of positive tests by month.

During January 1, 2021–January 15, 2022, of 169,908 samples tested for SARS-CoV-2 from children and adolescents of all ages, 15,320 (9.02%) were positive by an NAAT assay. (Figure 1). The monthly rate of SARS-CoV-2 NAAT positive tests fluctuated throughout the study, from ≈10.00% in January 2021, when the nonvariant strain predominated, to 3.78%–1.70% during March–June 2021, coinciding with the circulation of the Alpha variant (p = 0.01). After June there was a steady increase in SARS-COV-2 positivity rates when Delta predominated. The highest positivity rate of 33.05% was reached in January 2022 with the circulation of Omicron (Figure 1).

We performed variant screening on 1,058 (6.91%) positive samples for SARS-CoV-2, confirming the local circulation of 12 variants. Of those samples, 11.34% (120) corresponded to the nonvariant strain, 11.81% to Alpha, 62.77% to Delta, and 10.49% to Omicron. Thirty-eight patients (3.59%) were infected with other variants, including Beta, Gamma, Lota, Zeta, Eta, Epsilon, and Mu, as well as a single variant of uncertain importance.

### Demographic Characteristics of the Clinical Cohort

**Figure 2:**
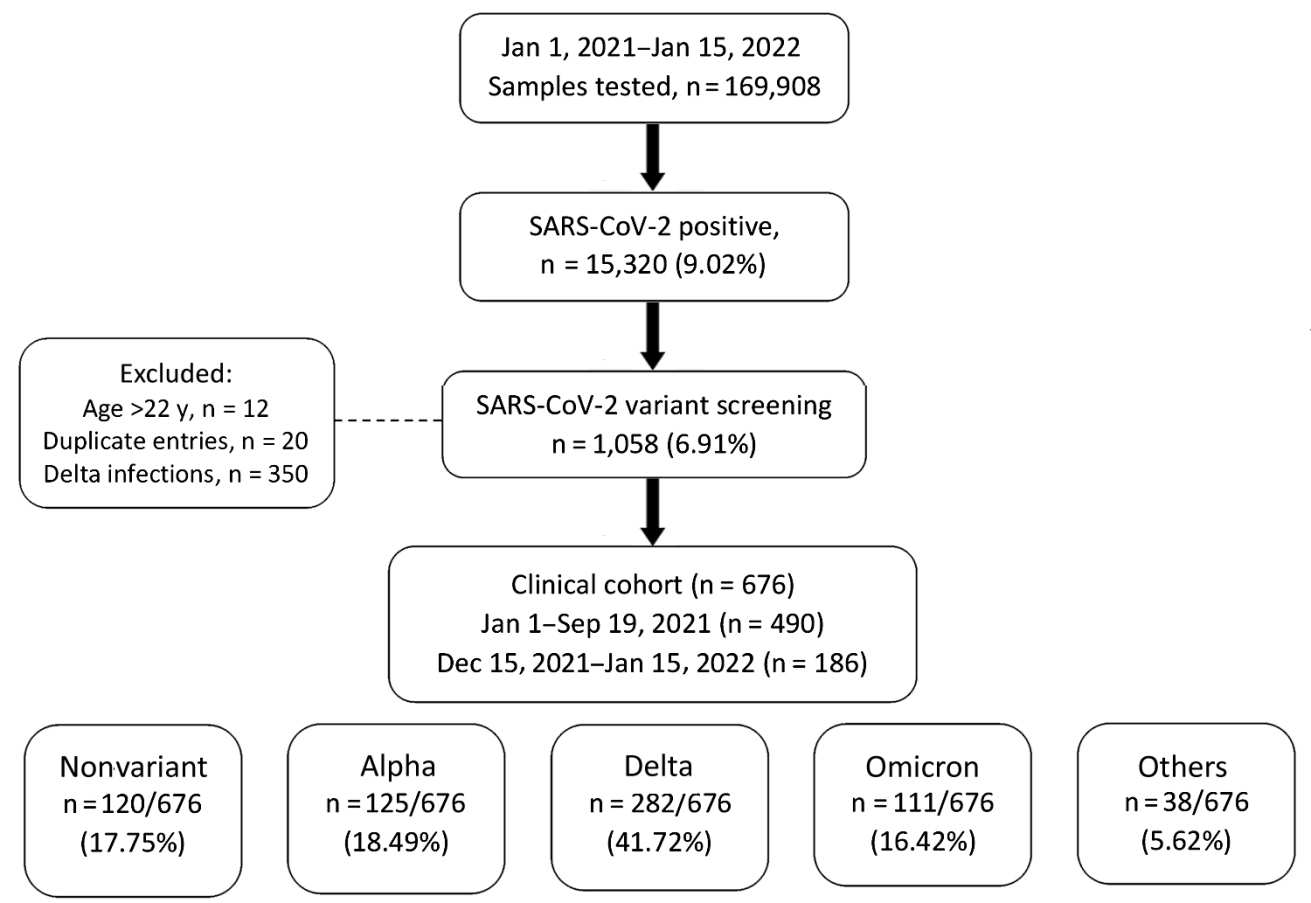
Flow diagram of sample and patient selection for SARS CoV-2 variant screening of nasopharyngeal samples at Nationwide Children’s Hospital, Columbus, Ohio, USA, during January 1, 2021–January 15, 2022. After excluding patients >22 years of age and duplicate entries, 676 patients with positive SARS-CoV-2 tests during January 1–September 19, 2021, and December 15, 2021–January 15, 2022 were included in the clinical analyses. Other variants were Beta, Iota, Zeta, Eta, Epsilon, Gamma, Mu, and other variants under investigation.

We included in final analyses sample data from 676 (63.89%) unique patients, comprising the clinical cohort (Table 1; Figure 2). Of the 676 patients, those identified during January 1–September 19, 2021, corresponded to nonvariant (n = 120, 17.75%), Alpha (n = 125, 18.49%), and Delta infections (n = 282, 41.72%). Patients identified during December 15, 2021– January 15, 2022, corresponded to Omicron B.1.1.529 infections (n = 111, 16.42%). Patients identified during September 20–December 14, 2021, corresponded to Delta infections but were not included because sample size for Delta infections was deemed sufficient.

**Table 1:** Demographic characteristics of the clinical cohort in study of SARS-CoV-2 variants in children and adolescents, Columbus, Ohio, USA*

Clinical cohort characteristics	All patients, n = 676	Outpatients, n = 450	Inpatients, n = 226	p value
Median age, y (IQR)	8.98 (2.64–14.71)	9.40 (3.90–14.23)	6.55 (0.48–15.60)	
Age group, y				
<1	102 (15.09)	32 (7.11)	70 (30.97)	**<0.001**
1–4	129 (19.08)	96 (21.33)	33 (14.60)	
5–11	189 (27.96)	157 (34.89)	32 (14.16)	
12–21	256 (37.87)	165 (36.67)	91 (40.27)	
Sex				
M	356 (52.66)	230 (51.11)	126 (55.75)	0.25
F	320 (47.34)	220 (48.49)	100 (44.25)	
Race/ethnicity	n = 587	n = 365	n = 222	0.36
White	349 (59.45)	215 (58.90)	134 (60.36)	
Black	146 (24.87)	92 (25.21)	54 (24.32)	
Multiracial	38 (6.47)	26 (7.12)	12 (5.41)	
Hispanic	37 (6.30)	25 (6.85)	12 (5.41)	
Other	17 (2.90)	7 (1.92)	10 (4.51)	
Underlying conditions†	n = 538	n = 312	n = 226	**<0.001**
None	288 (53.53)	198 (63.46)	90 (39.82)	
Yes	250 (46.47)	114 (36.54)	136 (60.18)	
Obesity/overweight	141 (26.21)	68 (21.79)	73 (32.30)	
SARS-CoV-2 vaccination status	n = 565	n = 361	n = 204	0.62
Received >1 dose	17 (3.01)	12 (3.32)	5 (2.45)	
Not immunized	548 (96.99)	349 (96.68)	199 (97.55)	
SARS-COV-2 variant				
Nonvariant	120 (17.75)	88 (19.56)	32 (14.16)	**<0.001**
Alpha	125 (18.49)	98 (21.78)	27 (11.95)	
Delta	282 (41.72)	189 (42.00)	93 (41.15)	
Omicron	111 (16.42)	49 (10.89)	62 (27.43)	
** Other‡**	38 (5.62)	26 (5.78)	12 (5.31)	

*Values are no. (%) except as indicated. Continuous variables were analyzed by Mann-Whitney test. Categorical data were analyzed by Fisher exact test or χ^2^ test. Bold indicates significance. IQR, interquartile range. †Underlying conditions included respiratory (asthma, bronchopulmonary dysplasia, cystic fibrosis); cardiac (septal defects, valvopathies; dilated cardiomyopathy); endocrinologic (diabetes, dyslipidemia, panhypopituitarism, polyendocrinopathy); hematologic (sickle cell disease, spherocytosis, thalassemia, chronic idiopathy thrombocytopenia purpura); immunocompromised conditions (solid or hematopoietic organ transplantation, malignancies–leukemias, solid tumors, rheumatologic conditions receiving chronic immunosuppressive therapy, primary immunodeficiencies, HIV); neurologic and genetic conditions (cerebral palsy, autism, developmental delay); prematurity; renal/gastrointestinal conditions (Hirschsprung’s, duodenal atresia, short bowel syndrome, chronic kidney disease, intestinal bowel disease, nephrotic syndrome), and obesity/overweight. ‡Other variants: Gamma (n = 12); Epsilon (n = 3); Eta (n = 2); Beta (n = 1); Iota (n = 9); Mu (n = 2); variants under investigation, (n = 2); Zeta (n = 7).

**Figure 3:**
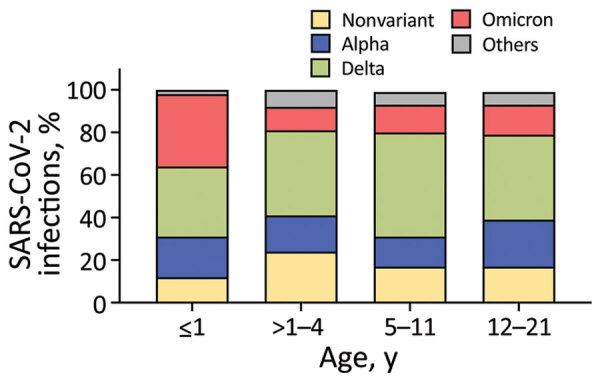
Distribution of SARS-CoV-2 variants pediatric and adolescent patients included in a clinical cohort (n = 676) at Nationwide Children’s Hospital, Columbus, Ohio, USA, by age group. Bars represent the percentage of each SARS-CoV-2–specific variant in the different age groups.

Of the 676 patients, we tested 450 (66.57%) as outpatients and 226 (33.43%) in the hospital. Median age for inpatients (6.6 [IQR 0.5–15.6] years) was lower than that for outpatients (9.4 [IQR 3.9–14.2] years; p<0.01). In both settings, infections were more common in adolescents 12–21 years of age, whereas in inpatients, infants were the second most common age group represented (30.97%). We observed no differences in sex and race/ethnicity between inpatients and outpatients. SARS-CoV-2 vaccination rates were low (3.01%) and did not differ between outpatients and inpatients either. Overall, Delta infections were the most common infections in inpatients (41.15%) and outpatients (42.00%). Alpha infections were more common in outpatients (21.78% vs. 11.95% in inpatients) and Omicron in inpatients (27.43% vs. 10.89% in outpatients). (Figure 3; Appendix Table 1).

Of the COVID-19 clinical cohort, 80.0% (538/676) had available data regarding underlying conditions. Underlying conditions were more prevalent in inpatients (60.18%) than in outpatients (36.54%; p<0.001); obesity/overweight was the most common. Compared with the overall population evaluated at NCH during the same period (NCH cohort; n = 444,425), the prevalence of complex chronic conditions identified by the PMCA algorithm was greater in the COVID-19 clinical cohort (31.56%) than in the overall NCH cohort (16.95%), whereas noncomplex chronic conditions were more common in the NCH cohort (20.76%) than in the clinical cohort (14.52%) (Appendix Table 2).

### Clinical Cohort Viral Loads and Viral Co-infections

**Figure 4:**
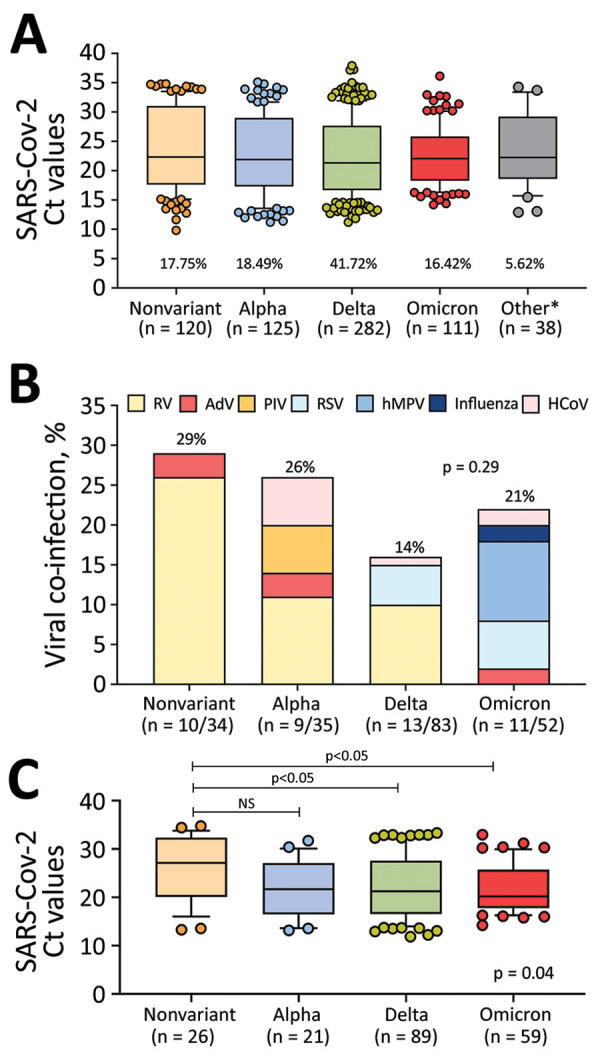
SARS-CoV-2 viral loads and viral co-infections among children and adolescents with COVID-19 at Nationwide Children’s Hospital, Columbus, Ohio, USA, by the infecting SARS-CoV-2 variant, January 1, 2021–January 15, 2022. A) Nasopharyngeal SARS-CoV-2 viral loads expressed as Ct values according to the infecting SARS-CoV-2 variant in the clinical cohort (n = 676). Percentage of total infections for each variant is below each bar. B) Viral co-infections by SARS-CoV-2 variant during the study period in patients that underwent multiplex viral testing. Twelve patients with other variants tested negative for viral co-infections (not shown). Percentage of total co-infections is above each bar. p value was determined by χ^2^ test. C) Nasopharyngeal SARS-CoV-2 Ct values by infecting SARS-CoV-2 variant among inpatients with acute COVID-19, excluding patients with MIS-C, SARS-CoV-2 detected by screening in inpatients, and those infected with uncommon SARS-CoV-2 strains. p value at bottom right represents the overall Kruskal-Wallis p value; values above bars indicate ad hoc pairwise comparisons by Dunn multiple test correction. For box plots in panels A and C, horizontal lines within boxes indicate medians; box tops and bottoms indicate interquartile ranges; error bars indicate 95% CIs. AdV, adenovirus; HCoV, human coronavirus; hPMV, human metapneumovirus; MIS-C, multisystem inflammatory syndrome in children; NS, not significant. PIV, parainfluenza virus; RSV, respiratory syncytial virus; RV, rhinovirus.

We assessed differences in SARS-CoV-2 Ct values in the clinical cohort according to the infecting variant and found comparable values (p = 0.35) (Figure 4, panel A). For 32.10% (217/676) of patients, we performed a multiplex respiratory viral panel; we identified SARS-CoV-2/viral coinfections in 43 patients (19.82%) (Figure 4, panel B). Rhinovirus/enterovirus (RV/EV) was the most common viral coinfection (n = 22) followed by respiratory syncytial virus (RSV; n = 7), human metapneumovirus (hMPV; n = 5), endemic coronavirus (n = 3), parainfluenza viruses (PIVs; n = 3), adenovirus (n = 3), and influenza viruses (n = 1). We observed no differences in the rates of co-infections according to the SARS-CoV-2 variant (p = 0.29). However, the type of viral coinfection varied throughout the study; RSV, hMPV, and influenza co-infections were identified only in children with Delta and Omicron infections.

### Clinical Characteristics of the Inpatient Cohort

Because complete clinical and laboratory data were available for inpatients (n = 226), we further analyzed this cohort. We excluded 21 patients, 11 with MIS-C, because this condition represents a postacute complication of COVID-19, and 10 who were diagnosed by screening upon admission, leaving a total of 205 inpatients with acute COVID-19. Of the 11 patients with MIS-C (median age 10.40 [IQR 2.10–15.70] years), 4 infections were related to the nonvariant strain, 2 to the Alpha variant, 3 to Delta, and 2 to Omicron. The 10 inpatients identified by SARS-CoV-2 screening were hospitalized with other infectious processes (i.e., rotavirus or *Clostridioides difficile* enteritis, intraabdominal abscesses, periorbital cellulitis) or trauma-related diagnoses.

Of the 205 inpatients with acute COVID-19, a total of 26 (12.68%) were infected with the nonvariant strain, 21 (10.24%) with Alpha, 89 (43.41%) with Delta, and 59 (28.78%) with Omicron. Ten patients were infected with other SARS-CoV-2 variants (Epsilon, Eta, Gamma, and Iota); given their low representation, they were excluded from further analyses, leaving 195 inpatients for comparative clinical analyses.

Inpatients infected with Omicron were significantly younger (0.88 [IQR 0.13–11.72] years) than those infected with Delta (11.11 [IQR 0.69–16.05] years; p<0.001). Almost half (46.07%) of inpatients with Delta infections were adolescents, whereas infants represented 52.54% of Omicron infections (Table 2). Most inpatients in all variant groups were White, except inpatients with Alpha infections, who were mostly Black (57.14%). Underlying conditions were prevalent (61.03%); obesity/overweight was the most common chronic comorbidity irrespective of the infecting variant. Most (97.44%) inpatients were not immunized against SARS-CoV-2.

**Table 2:** Demographic, laboratory characteristics and clinical outcomes of children and adolescents hospitalized with acute COVID-19 by SARS-CoV-2 variant, Columbus, Ohio, USA*

Characteristic	Clinical inpatient cohort, n = 195†	Nonvariant, n = 26	Alpha, n = 21	Delta, n = 89	Omicron, n = 59	p value
Median age, y (IQR)	5.7 (0.36–15.41)	3.3 (1.25–15.46)	4.1 (0.48–14.84)	11.1 (0.69–16.05)	0.8 (0.13–11.72)	**0.01‡**
Age group, y						**<0.001‡**
<1 y	67 (34.36)	5 (19.23)	8 (38.10)	23 (25.84)	31 (52.54)	
1–4	27 (13.85)	10 (38.46)	3 (14.29)	8 (8.99)	6 (10.17)	
5–11	27 (13.85)	1 (3.85)	2 (9.52)	17 (19.10)	7 (11.87)	
12–21	74 (37.95)	10 (38.46)	8 (38.07)	41 (46.07)	15 (25.42)	
Sex						
M	106 (54.36)	15 (57.69)	10 (47.62)	50 (56.18)	31 (52.54)	0.89
F	89 (45.64)	11 (42.31)	11 (52.38)	39 (43.82)	28 (47.46)	
Race/ethnic group						**0.02‡**
White	113 (57.95)	18 (69.23)	8 (38.10)	59 (66.30)	28 (47.46)	
Black	46 (23.59)	4 (15.39)	12 (57.14)	16 (17.98)	14 (23.73)	
Multiracial	11 (5.64)	2 (7.69)	1 (4.77)	4 (4.49)	4 (6.78)	
Hispanic	11 (5.64)	1 (3.85)	0 (0.00)	5 (5.62)	5 (8.48)	
Other/unknown	14 (7.18)	1 (3.85)	0 (0.00)	5 (5.62)	8 (13.56)	
Underlying conditions§	119 (61.03)	17 (65.38)	14 (66.67)	58 (65.19)	30 (51.72)	0.3
Obesity/overweight	63 (32.30)	6 (23.08)	6 (28.57)	38 (42.70)	13 (22.03)	
Respiratory	21 (10.77)	3 (11.54)	6 (28.57)	10 (11.24)	2 (3.39)	
Genetic/neurologic	25 (12.82)	4 (15.38)	1 (4.76)	11 (12.36)	9 (15.25)	
Cardiac	5 (2.56)	3 (11.54)	0 (0.00)	1 (1.12)	1 (1.69)	
GI/renal	10 (5.13)	3 (11.54)	1 (4.76)	6 (6.74)	0 (0.00)	
Other	4 (24.10)	5 (19.23)	5 (23.81)	20 (22.47)	17 (28.81)	
SARS-CoV-2 vaccination	5 (2.82)	0 (0.00)	0 (0.00)	1 (1.13)	4 (6.78)	0.27
Duration of illness, d (IQR)	3 (1.00–7.00)	3 (1.00–6.00)	2 (1.00–5.00)	5 (2.00–8.00)	2 (1.00–4.00)	**<0.001‡**
Clinical manifestations						
Fever	147 (75.38)	14 (53.85)	16 (76.19)	71 (79.78)	46 (77.97)	**<0.05‡**
Respiratory	160 (82.05)	16 (61.54)	17 (80.95)	75 (84.27)	52 (88.14)	**0.03‡**
Upper respiratory	70 (43.75)	6 (23.08)	8 (38.10)	28 (31.46)	28 (47.46)	0.11
Lower respiratory	90 (56.25)	10 (38.46)	9 (42.86)	47 (52.81)	24 (40.68)	0.39
Cardiac	25 (12.82)	5 (19.23)	3 (14.29)	12 (13.48)	5 (8.48)	0.57
Gastrointestinal	83 (42.56)	11 (42.31)	9 (42.86)	36 (40.45)	27 (45.77)	0.94
Other¶	47 (24.10)	9 (34.62)	12 (57.14)	28 (31.46)	20 (33.90)	0.17
ALC, × 10^3^/μL	1.6 (0.93–3.46)	2.2 (1.62–3.69)	1.90 (1.26–3.36)	1.20 (0.87–2.06)	2.7 (0.93–4.38)	**0.01‡**
Lymphopenia	105/170 (61.76)	9 (37.50)	13 (61.91)	57 (72.15)	26 (56.52)	**0.02‡**
CRP, mg/dL, median (IQR)	1.4 (0.50–4.10)	2.50 (0.65–5.75)	0.70 (0.50-.3.40)	1.70 (0.55–3.75)	1.30 (0.50–4.40)	0.62
Ct, median (IQR)	21.36 (17.28–27.69)	27.10 (20.16–31.98)	21.67 (16.64–26.35)	21.24 (16.54–27.35)	20.17 (17.80–25.26)	**0.04‡**
COVID-19 targeted therapy	70 (35.90)	6 (23.08)	7 (33.33)	38 (42.70)	19 (32.20)	0.26
Oxygen supplementation	91 (46.67)	10 (38.46)	9 (42.86)	50 (56.18)	22 (37.29)	0.1
PICU admission	53 (27.18)	7 (26.92)	5 (23.81)	26 (29.21)	15 (25.42)	0.94
Duration of PICU stay, d (IQR)	3.48 (1.00–7.46)	1.00 (1.00–2.50)	3.00 (2.00–34.00)	4.00 (1.63–7.65)	4.00 (2.03–5.44)	0.26
Duration of hospitalization, d (IQR)	2.86 (1.79–7.09)	2.10 (1.72–3.02)	2.89 (1.81–5.00)	3.91 (1.84–7.91)	2.20 (1.45–7.33)	0.17

*Values are no. (%) except as indicated. Continuous variables were analyzed by analyzed by Kruskal-Wallis test. Categorical data were analyzed by χ^2^ test. Bold text indicates statistical significance. ALC, absolute lymphocyte count; CRP, C-reactive protein; GI, gastrointestinal; IQR, interquartile range; PICU, pediatric intensive care unit. †Patients with multisystem inflammatory syndrome in children, SARS-CoV-2 detected by screening but hospitalized for other reasons, and those infected with uncommon SARS-CoV-2 strains were excluded from analyses. ‡p values represent statistical significance between groups based on Kruskal-Wallis or χ^2^ test. Ad hoc adjusted p values from pairwise comparisons were as follows: age, p<0.01 between Omicron and Delta; duration of illness, Delta vs. nonvariant infections (p = 0.03), Delta vs. Alpha (p not significant); and Delta vs. Omicron (p<0.001); fever, nonvariant vs. Delta (p = 0.01), nonvariant vs. Omicron (p = 0.04); respiratory symptoms, nonvariant vs. Delta (p = 0.03), nonvariant vs Omicron (p < 0.01). Total ALC, Delta vs. nonvariant infections (p = 0.03); lymphopenia, Delta vs. nonvariant infections (p<0.01). Ct values: nonvariant vs. Delta (p = 0.03), nonvariant vs Omicron (p = 0.03). §Underlying conditions: respiratory (asthma, bronchopulmonary dysplasia, cystic fibrosis); cardiac (septal defects, valvopathies; dilated cardiomyopathy); endocrinologic (diabetes, dyslipidemia, panhypopituitarism and polyendocrinopathy); hematologic (sickle cell disease, spherocytosis, thalassemia, chronic idiopathy thrombocytopenia purpura); immunocompromised conditions (solid or hematopoietic organ transplantation, malignancies–leukemias, solid tumors, rheumatologic conditions receiving chronic immunosuppressive therapy, primary immunodeficiencies, HIV); neurologic and genetic conditions (cerebral palsy, autism, developmental delay); prematurity; renal/gastrointestinal conditions (Hirschsprung’s, duodenal atresia, short bowel syndrome, chronic kidney disease, intestinal bowel disease, nephrotic syndrome); and obesity/overweight. ¶Other symptoms: anosmia, fatigue, myalgias, headache, back pain, chills, polyarthralgia, hemoptysis, rash. Lymphopenia defined as ALC of <4,500 cells/μL in infants, and <1,500 cells/μL in children >12 mo of age (*18*).

Duration of symptoms at the time of the SARS-CoV-2 testing was longer in inpatients with Delta infections than in those with nonvariant and Omicron infections (p<0.001), yet inpatients with Delta and Omicron infections had significantly lower Ct values than did those infected with the nonvariant strain (p = 0.04) (Figure 4, panel C). Compared with those with nonvariant infections, inpatients with Delta and Omicron infections were brought for care with fever and respiratory symptoms more frequently (p<0.05). Absolute lymphocyte counts (ALC) and lymphopenia, defined as an ALC of <4,500 cells/μL in children <12 months and <1,500 cells/μL in children >12 months of age (*18*), were more common in inpatients with Delta infections (p = 0.01) than those with nonvariant strain infections (p = 0.02).

COVID-19 therapy was provided to 42.70% of inpatients with Delta infections compared with 23.08% of those with nonvariant infections or 33.33% of inpatients infected with the Alpha variant, with no differences between groups (p = 0.26). Fifty-six percent of inpatients with Delta infections received oxygen, compared with ≈40.00% of those with other variants. Intensive care unit (ICU) admission was required for ≈25% of inpatients irrespective of the infecting variant; however, inpatients with Delta infections stayed in the ICU and in the hospital for a median of 1–2 days longer than those with other variants. These differences did not reach statistical significance.

One patient who had morbid obesity and acute COVID-19 associated with the Alpha variant died. In addition, 12 patients had more severe or unusual clinical manifestations: 6 were nonvaccinated patients 8–20 years of age with Delta infections whose illness manifested with severe myocarditis, pulmonary embolism, pneumothorax, or pneumomediastinum, and the other 6 were children <3 years of age with Omicron infections that manifested as croup.

### Evaluation of Risk Factors for Severe COVID-19

We performed multivariable analyses to identify risk factors associated with disease severity defined as need for hospitalization, administration of supplemental oxygen, and PICU admission in patients with acute COVID-19. Fifteen children received mechanical ventilation, which precluded further multivariable analyses. Presence of underlying chronic conditions (odds ratio [OR] 4.53, 95% CI 1.48–15.10) and infants (OR 6.64, 1.34–36.00), but not the infecting SARS-CoV-2 variant or viral co-infections, were independently associated with increase odds of hospitalization (Appendix Table 3). In hospitalized patients, underlying conditions also increased the odds for supplemental oxygen administration (OR 2.62 95% CI 1.01–6.95); viral co-infections increased the odds, but the difference was not significant (OR 2.75, 95% CI 0.98–8.17; p = 0.06) (Appendix Table 4). In addition, OR for PICU admission was higher in inpatients with SARS-CoV-2/viral co-infections (OR 2.89, 95% CI 1.03–8.99) (Appendix Table 5).

## Discussion

The emergence of distinct SARS-CoV-2 variants since the beginning of the COVID-19 pandemic and the questionable differences in severity among variants in children remains poorly understood. To date, most of the studies describing the clinical effects of SARS-CoV-2 variants have been conducted in adults or derived from national or regional estimates, without a direct nexus between the specific SARS-CoV2 variant and the patient’s clinical phenotype (*19*–*22*). In this study, we linked the PCR-identified SARS-CoV-2 variant and patient clinical characteristics. We found that children and adolescents hospitalized for acute Delta and Omicron infections had lower SARS-CoV-2 Ct values and experienced fever and respiratory symptoms more frequently than did inpatients infected with previous variants. In adjusted analyses, presence of underlying conditions and viral co-infections, but not the infecting variant, were associated with worse clinical outcomes. Overall, these data suggested that different SARS-CoV-2 variants are associated with distinct clinical manifestations; however, clinical risk factors remain important determinants of COVID-19 severity.

We documented the local circulation of 12 SARS-CoV-2 variants appearing temporally in waves that coincided with national reports (*19*). First, the Alpha variant circulated until June 2021, followed by Delta during July–December 2021, and more recently Omicron. In our study, the highest positivity rate for SARS-CoV-2 occurred in January 2022, when Omicron predominated, which mirrors findings of national reports (*23*,*24*) and supports the high transmissibility of this variant (*25*,*26*). The rates and pattern of viral co-infections with SARS-CoV-2 in our cohort are similar to described previously (*27*–*29*). Whereas rhinovirus/enterovirus was the most common viral coinfection identified throughout the study, coinfections with enveloped viruses (coronavirus, RSV, PIV, hMPV, or influenza) increased as nonpharmacologic interventions to prevent SARS-COV-2 infections were discontinued (*30*). The atypical RSV season documented in summer 2021 (*31*,*32*) coinciding with the Delta wave was also evident in our cohort; most children with RSV/SARS-CoV-2 co-infections were identified during July–December 2021.

We observed a U-shaped age distribution of COVID-19 in children, which was previously reported (*33*,*34*). Within all age groups, patients 12–21 years of age were 37.87% of all patients and 40.27% of those who were hospitalized, especially with Delta, Alpha, and nonvariant infections. On the other hand, half of Omicron infections were documented in infants, compared with 20%–30% of infant infections with previous variants. These findings are consistent with other US and UK studies that reported an increased proportion of infants and young children hospitalized with COVID-19 during the Omicron wave (*23*,*24*). In our cohort, although the predominant race was White (59%), a significant number of Black and Hispanic children were affected irrespective of the infecting variant, confirming previous studies (*29*,*35*). Obesity has been consistently associated with severe COVID-19 in adults (*35*–*37*); we found that in children, obesity/overweight was the underlying condition most commonly associated with worse clinical outcomes irrespective of the infecting variant. Almost none of the children hospitalized with COVID-19 were vaccinated, reflecting national trends (*23*,*38*).

Studies conducted in adults suggested that infections with the Delta variant were associated with more severe disease and higher viral loads than infections with previous variants (*25*,*39*–*45*). On the other hand, subsequent reports using national US trends or EHR data not linked to specific variants showed that disease severity in children during the Delta wave was comparable to that described with the circulation of previous variants (*19*,*22*). In our study we found that PCR-typed Delta infections were associated with lower Ct values, more frequent fever and respiratory symptoms, and higher rates of lymphopenia than infections caused by the original strain. Moreover, a great proportion of children and adolescents with severe manifestations were infected with the Delta variant. On the other hand, children infected with Omicron were younger than in previous waves and had lower Ct values; nearly half (47.46%) experienced upper respiratory symptoms including croup, which was anecdotally reported earlier in the pandemic (*46*,*47*). Although information about preexisting antibodies or other host factors was not available in these children, the differences in clinical manifestations by variant might partially reflect the evolution and fitness of SARS-CoV-2 associated with differences in transmissibility or pathogenicity.

A recent retrospective study conducted in children <5 years of age with COVID-19 showed that those identified during the Omicron surge were younger and had a lower risk for severe disease than children identified during the Delta wave (*48*). Similarly, another large retrospective study showed that rates of hospitalization in US children 0–4 years of age during the initial wave of Omicron (late December 2021–February 2022) were 5 times higher than with the circulation of Delta, yet clinical disease severity was worse during the Delta wave (*23*,*24*). Contrary to those studies, we found that rates of PICU admission were similar between children with Omicron and those infected with all other variants. We also found that a higher proportion of RSV and hMPV/SARS-CoV-2 co-infections were identified in children with Omicron and that SARS-CoV-2/viral co-infections were associated with increased odds of PICU admission. Our study is likely underpowered to determine whether it is plausible that RSV or hMPV co-infections could have played a role in the higher rates of PICU admission observed in children with Omicron infection.

One patient in our study who was infected with the Alpha variant and with multiple chronic conditions died. Although death associated with COVID-19 in children is low, >1,400 children and adolescents 0–18 years of age have died of COVID-19 in the United States as of September 2022 (*49*,*50*).

The first limitation of our study is that not all samples that tested positive for SARS-CoV-2 by NAAT underwent variant screening. The percentage of monthly samples screened varied based upon sample volumes and the availability of the personnel at the NCH clinical laboratory. Therefore, during months of high SARS-CoV-2 activity, a smaller percentage of samples underwent variant testing. In addition, clinical data from 350 patients with Delta infections identified during September–December 2021 were not collected because we had a sufficient sample size for Delta infections. Although the cohort we analyzed was a convenience sample, it is representative of the overall population evaluated in our center during the pandemic. Another limitation is related to the retrospective nature of data collection, which affected the outpatient cohort. We reviewed all patient records manually, but data regarding clinical manifestations or duration of symptoms were not available for all outpatients. Thus, to mitigate the impact of missing data, we analyzed clinical variables exclusively in inpatients.

In summary, our findings confirmed the local circulation of different SARS-CoV-2 variants over time infecting children and adolescents treated at a children’s hospital in Ohio, USA. Infections caused by Delta and Omicron variants were associated with lower Ct values and with more frequent fever and respiratory symptoms than for infections with the original strain; at least one fourth of hospitalized children required ICU admission, irrespective of the infecting variant. These findings suggest that children are susceptible to SARS-CoV-2 infection by any of the circulating variants and that they can develop severe disease. The data also emphasize that active monitoring of the shift in SARS-CoV-2 variants is critical to understand their clinical effects and implications for managing COVID-19 in children.

Appendix Additional information about differences in SARS-CoV-2 clinical manifestations and disease severity in children and adolescents by SARS-CoV-2 variant.
