# Supplementary material for: Differences in SARS-CoV-2 Clinical Manifestations and Disease Severity in Children and Adolescents by Infecting Variant
Source: Emerg Infect Dis. 2022 Nov;28(11):2270–80. doi: 10.3201/eid2811.220577 (PMC9622241; doi:10.3201/eid2811.220577)
Supplement: Appendix — Additional information about differences in SARS-CoV-2 clinical manifestations and disease severity in children and adolescents by SARS-CoV-2 variant. [file 22-0577-Techapp-s1.pdf]

Article DOI: https://doi.org/10.3201/eid2811.220577

Differences in SARS-CoV-2 Clinical Manifestations and Disease Severity in Children and Adolescents by the Infecting Variant

Appendix

SARS-CoV-2 Testing at NCH

The nucleic acid amplification tests (NAATs) for SARS-CoV-2 detection used by the Clinical Microbiology Laboratory at Nationwide Children's Hospital (NCH) per standard of care were included the BioFire FilmArray Respiratory Panel RP2.1 (BioFire, https://www.biofiredx.com), Xpert XpressCoV-2 PCR (Cepheid, https://www.thermofisher.com), Hologic SARS-CoV-2 assay (https://www.hologic.com), BioGX SARS-CoV-2 assay (https://www.biogx.com), and the CDC-modified PCR assay developed at NCH (*1*).

SARS-CoV-2 Delta Variant Identification

Briefly, 5 µL of the total nucleic acid eluate was added to a 20-µL total-volume reaction mixture (1× TaqPath 1-Step RT-qPCR Master Mix, CG [Thermo Fisher Scientific, https://www.thermofisher.com]), with 0.9 µM each primer (S-T478K F: 5′-TGAGAGAGATATTTCAACTGAAATCTATCAG-3′ and S-T478K R: 5′-AGGAAAGTAACAATTAAAACCTTAACACCA −3′) and 0.2 µM each probe (S-T478 P1: 5′-FAM- CCGGTAGCAcACCT –MGB-3′and S-T478K P2: 5′-VIC- CGGTAGCAaACCT –MGB-3′). The RT-PCR assay was analyzed in the ABI 7500 thermocycler (Life Technologies, https://www.thermofisher.com). The reaction was carried out under the following running conditions: 25°C for 2 min, then 50°C for 15 min, followed by 10 min at 95°C and 45 cycles of 95°C for 15 s and 65°C for 1 min. Samples displaying typical amplification curves above the threshold were considered positive.

Appendix Table 1. Clinical cohort by age group and infecting SARS-CoV-2 variant, Nationwide Children's Hospital, Columbus, Ohio, USA, January 1, 2021–January 15, 2022*

Infecting variant	Infants, n = 102 (15.09%)	1–4 y, n = 129 (19.08%)	5–11 y, n = 189, (27.96%)	12–21 y, n = 256 (37.87%)	p value
Nonvariant	12 (11.77%)	31 (24.03%)	33 (17.46%)	44 (17.19%)	
Alpha	19 (18.63%)	22 (17.05%)	27 (14.29%)	57 (22.27%)	
Delta	34 (33.33%)	52 (40.31%)	93 (49.21%)	103 (40.23%)	**<0.001**
Omicron	35 (34.31%)	14 (10.85%)	25 (13.23%)	37 (14.45%)	
Others†	2 (1.96%)	10 (7.75%)	11 (5.82%)	15 (5.86%)	

*Bold indicates significance. Categorical data analyzed by χ^2 test.
‡Other variants include: Beta, Iota, Zeta, Eta, variants of interest, Epsilon, Gamma and Mu.

Appendix Table 2. Contrast of race/ethnicity and underling chronic conditions between the COVID-19 clinical cohort and overall population 0–21 y of age evaluated during the study period, Nationwide Children's Hospital, Columbus, Ohio, USA, January 1, 2021–January 15, 2022*

Variables	COVID-19 clinical cohort, n = 676	NCH cohort, n = 444,425	p value
Race/ethnicity			**0.01**
White	359 (53.19%)	247,232 (55.63%)	
Black or African American	148 (21.93%)	99,875 (22.47%)	
Multiple race	45 (6.67%)	28,946 (6.51%)	
Asian	18 (2.67%)	16,674 (3.75%)	
Others/unknown	105 (15.53%)	50,793 (11.43%)	
Hispanic	31 (4.59%)	27,321 (6.15%)	
Underlying diseases (PMCA)			**<0.001**
None	364 (53.93%)	265,682 (59.78%)	
Noncomplex chronic	98 (14.52%)	92,243 (20.76%)	
Complex chronic	213 (31.56%)	75,339 (16.95%)	

*Categorical data analyzed by χ^2 test. PMCA, Pediatric Medical Complexity Algorithm.

Appendix Table 3. Adjusted odds for hospitalization in children and adolescents with acute COVID-19, Nationwide Children's Hospital, Columbus, Ohio, USA, January 1, 2021–January 15, 2022*

		Univariable analyses		Multivariable analyses	
Variables		OR (95% CI)	p value	RR (95% CI)	p value
Age groups†					
Infants	62 (34.62%)	3.42 (0.93–13.10)	0.06	6.64 (1.34–36.00)	**0.02**
1–4 y	31 (17.32%)	4.35 (0.90–31.8)0	0.09	5.39 (0.85–49.00)	0.09
12–21 y	60 (33.52%)	1.34 (0.41–4.03)	0.61	0.97 (0.22–3.77)	0.96
Variants‡					
Alpha	26 (14.53%)	0.54 (0.14–1.90)	0.34	0.66 (0.15–2.83)	0.58
Delta	74 (41.34%)	1.72 (0.48–5.57)	0.38	2.49 (0.61–9.86)	0.19
Omicron	53 (29.61%)	6.07 (1.21–44.8)	**0.04**	5.95 (1.02–48.80)	0.06
Low Ct values§	96 (53.63%)	0.53 (0.21–1.29)	0.17	0.32 (0.09–0.93)	**0.04**
Viral co-infections	35 (19.55%)	0.54 (0.21–1.49)	0.21	0.37 (0.11–1.23)	0.10
Underlying conditions	96 (53.63%)	1.74 (0.74–4.28)	0.21	4.53 (1.48–15.10)	**<0.01**

*Odds of hospitalization was assessed in the cohort of patients that underwent multiplex viral testing (n = 179). Patients with MIS-C, SARS-CoV-2 detected by screening but hospitalized for other reasons, and patients infected with uncommon SARS-CoV-2 strains were excluded from analyses. Bold text indicates statistical significance. Ct, cycle threshold; OR, odds ratio; RR; risk ratio,
†Reference for age groups is 5–11 y of age.
‡Reference for variants is the nonvariant.
§Ct values were dichotomized by the median Ct values for each variant (Alpha, 23.1; Delta, 21.9; Omicron 20.3 Ct). High Ct values were used as a reference.

Appendix Table 4. Adjusted odds of oxygen supplementation in inpatients with acute COVID-19, January 1, 2021–January 15, 2022*

Characteristic	n = 140†	Univariable analyses		Multivariable analyses	
		OR (95% CI)	p value	OR (95% CI)	p value
Age group‡					
Infants	56 (40.00%)	0.20 (0.06–0.61)	**<0.01**	0.48 (0.11–1.95)	0.31
1–4 y	24 (17.14%)	0.27 (0.07–0.95)	**0.05**	0.39 (0.09–1.63)	0.20
12–21 y	42 (30.00%)	0.77 (0.21–2.51)	0.67	1.08 (0.28–3.81)	0.91
Variants§					
Alpha	15 (10.71%)	0.77 (0.18–3.23)	0.72	0.69 (0.14–3.32)	0.65
Delta	62 (44.29%)	1.13 (0.36–3.55)	0.83	1.03 (0.28–3.73)	0.96
Omicron	48 (34.29%)	0.63 (0.19–2.01)	0.43	0.63 (0.17–2.35)	0.50
Low Ct values¶	80 (57.14%)	1.00 (0.51–1.96)	1.00	1.19 (0.55–2.60)	0.66
Viral co-infections	25 (17.86%)	1.64 (0.96–4.05)	0.27	2.75 (0.98–8.17)	0.06
Underlying conditions	80 (57.14%)	3.79 (1.89–7.85)	**<0.01**	2.62 (1.01–6.95)	**0.04**

*Odds of oxygen supplementation was assessed in the cohort of inpatients with acute COVID-19 that underwent multiplex viral testing (n = 140). Patients with MIS-C, SARS-CoV-2 detected by screening but hospitalized for other reasons, and patients infected with uncommon SARS-CoV-2 strains were excluded from analyses. Bold indicates statistical significance. OR, odds ratio.
†Including inpatients with symptomatic COVID-19 infected with the nonvariant, Alpha, Delta or Omicron strains who had a viral respiratory panel performed.
‡Reference for age groups is 5–11 y.
§Reference for variants is the nonvariant.
¶Ct values were dichotomized by the median Ct values for each variant (Alpha, 23.1; Delta, 21.9; Omicron 20.3 Ct). High Ct values were used as a reference.

Appendix Table 5. Adjusted odds for PICU admission in inpatients with symptomatic COVID-19, January 1, 2021–January 15, 2022*

Characteristic	n = 140†	Univariable analyses		Multivariable analyses	
		OR [95% CI]	p value	OR [95% CI]	p value
Age groups‡					
Infants	56 (40.00%)	0.20 [0.06–0.60]	**<0.01**	0.35 [0.08–1.49]	0.16
1–4 y	24 (17.14%)	0.33 [0.09–1.16]	0.09	0.33 [0.07–1.39]	0.14
12–21 y	42 (30.00%)	0.66 [0.21–2.00]	0.46	0.86 [0.26–2.89]	0.81
Variants§					
Alpha	15 (10.71%)	0.42 [0.08–1.86]	0.26	0.37 [0.07–1.84]	0.23
Delta	62 (44.29%)	0.63 [0.20–2.01]	0.42	0.53 [0.14–1.95]	0.34
Omicron	48 (34.29%)	0.47 [0.14–1.57]	0.21	0.49 [0.13–1.88]	0.29
Low Ct values¶	80 (57.14%)	0.79 [0.39–1.60]	0.50	0.91 [0.41–2.00]	0.81
Viral co-infections	25 (17.86%)	2.11 [0.84–5.12]	0.10	2.89 [1.03–8.33]	**0.04**
Underlying conditions	80 (57.14%)	3.11 [1.47–6.94]	**<0.01**	2.01 [0.70–5.91]	0.20

*Odds of PICU admission was assessed in the cohort of inpatients with acute COVID-19 that underwent multiplex viral testing (n = 140). Patients with MIS-C, SARS-CoV-2 detected by screening but hospitalized for other reasons, and patients infected with uncommon SARS-CoV-2 strains were excluded from analyses. Bold indicates statistical significance. OR, odds ratio; PICU, pediatric intensive care unit.
†Including inpatients with symptomatic COVID-19 infected with the nonvariant, Alpha, Delta or Omicron strains who had a viral respiratory panel performed.
‡Reference for age groups is 5–11 y of age.
§Reference for variants is the nonvariant.
¶Ct values were dichotomized by the median Ct values for each variant (Alpha, 23.1; Delta, 21.9; Omicron 20.3 Ct). High Ct values were used as a reference.

Reference

1. Mertz C, Glowinski R, Cohen SH, Mertz S, Ye F, Hall MW, et al. Severe acute respiratory syndrome coronavirus 2 RNAemia and clinical outcomes in children with coronavirus disease 2019. J Infect Dis. 2022;225:208–13. https://doi.org/10.1093/infdis/jiab491
